# GigaTON: an extensive publicly searchable database providing a new reference transcriptome in the pacific oyster *Crassostrea gigas*

**DOI:** 10.1186/s12859-015-0833-4

**Published:** 2015-12-02

**Authors:** Guillaume Riviere, Christophe Klopp, Nabihoudine Ibouniyamine, Arnaud Huvet, Pierre Boudry, Pascal Favrel

**Affiliations:** Institute for fundamental and applied biology, University of Caen Basse-Normandie, Caen, France; UMR BOREA ‘Biologie des organismes et écosystèmes aquatiques’, Université de Caen Basse-Normandie, CNRS-7208, IRD-207, MNHN, UPMC, UCBN, UAG, Caen, F-14032 France; INRA, Sigenae UR875 Biométrie et Intelligence Artificielle, BP 52627, Castanet-Tolosan Cedex, 31326 France; Ifremer, UMR CNRS 6539 (CNRS/UBO/IRD/Ifremer), Laboratoire des Sciences de l’Environnement Marin, ZI de la Pointe du Diable, CS 10070, Plouzané, 29280 France

**Keywords:** Oyster, Transcriptome, Development, Stress, Tissues, NGS

## Abstract

**Background:**

The Pacific oyster, *Crassostrea gigas*, is one of the most important aquaculture shellfish resources worldwide. Important efforts have been undertaken towards a better knowledge of its genome and transcriptome, which makes now *C. gigas* becoming a model organism among lophotrochozoans, the under-described sister clade of ecdysozoans within protostomes. These massive sequencing efforts offer the opportunity to assemble gene expression data and make such resource accessible and exploitable for the scientific community. Therefore, we undertook this assembly into an up-to-date publicly available transcriptome database: the GigaTON (Gigas TranscriptOme pipeliNe) database.

**Description:**

We assembled 2204 million sequences obtained from 114 publicly available RNA-seq libraries that were realized using all embryo-larval development stages, adult organs, different environmental stressors including heavy metals, temperature, salinity and exposure to air, which were mostly performed as part of the *Crassostrea gigas* genome project. This data was analyzed *in silico* and resulted into 56621 newly assembled contigs that were deposited into a publicly available database, the GigaTON database. This database also provides powerful and user-friendly request tools to browse and retrieve information about annotation, expression level, UTRs, splice and polymorphism, and gene ontology associated to all the contigs into each, and between all libraries.

**Conclusions:**

The GigaTON database provides a convenient, potent and versatile interface to browse, retrieve, confront and compare massive transcriptomic information in an extensive range of conditions, tissues and developmental stages in *Crassostrea gigas*. To our knowledge, the GigaTON database constitutes the most extensive transcriptomic database to date in marine invertebrates, thereby a new reference transcriptome in the oyster, a highly valuable resource to physiologists and evolutionary biologists.

## Background

The Pacific Oyster, *Crassostrea gigas*, is a bivalve mollusk that belongs to lophotrochozoans, an under-described phylogenetic group among protostomes despite being the sister clade of ecdysozoans, which encompass insects and nematodes. *C. gigas* is an intertidal bivalve with a pelagic larval phase followed by a benthic adult life. Pacific oysters are farmed in intertidal or nearshore areas that undergo highly variable environmental conditions as well as an important anthropization, making *C. gigas* considered a sentinel species. Besides, *C. gigas* constitutes one of the most important shellfish aquaculture resources worldwide with an estimated market value reaching 1.3 billion dollars in 2013 (FAO). In this context, knowledge about the physiology of *C. gigas* is of fundamental, ecological and applied interests, leading to the generation of an important amount of transcriptomic data, as illustrated by the Gigasdatabase expressed-sequenced tags (EST) database [1]. These ESTs have enabled the development of microarrays used in physiological studies notably focusing on reproduction [2, 3], resistance to summer mortality [4–6], hypoxia [7], thermal stress [8] or ploïdy [9]. Additionally, MPSS or RNA-Seq analyses have been performed to investigate molecular processes involved in larval growth heterosis [10], immune responses to pathogenic bacteria [11] or response to salinity stress [12]. In many studies, biological understanding of data was hampered by limited annotation of transcripts, notably due to incomplete assembly or lack of reference transcriptome resource.

Aside from transcriptome studies, the genome of the Pacific oyster has recently been sequenced and assembled, and is now available for the community to explore [13]. The present assembly of the oyster genome has been annotated and genes were predicted *in silico* by comparison with other species. Together with the genome characterization, extensive transcriptomic data have also been generated from a very broad range of developmental stages and physiological conditions giving rise to 114 RNA-seq libraries. These data were directly mapped to the genome and used to gain insights into developmental, shell formation and stress response issues in the oyster from studies based on differential expression of the predicted genes.

Up to date, these RNA-seq data were mostly used to validate the published version of the genome assembly. In addition, such a massive sequencing effort [13] offers the opportunity to gain more insights into the transcriptome of the Pacific oyster at different development stages and under different environmental conditions. Insights into these features are critical for our understanding of many aspects of the biology in marine bivalves and their evolution. However, there is to date, to the best of our awareness, no convenient publicly accessible resource (i) where all the transcriptomic data is assembled and gathered and (ii) that is able to answer the need for a convenient way to browse into such an important amount of data. Therefore, we further exploited the *C. gigas* transcriptomic data generated in parallel to the genome characterization by Zhang et al [13], by per-sample assembling, then meta-assembling of the obtained contigs, the 2204 million reads from the 114 RNA-seq libraries. We provide these results including sequence of complete open reading frames (ORFs) (thereby protein), presence of transcript variants, knowledge of 5’ and 3’-untranslated regions, polymorphism and expression levels between physiological stages and conditions into an extensive, searchable, highly potent and user-friendly online database based on RNABrowse [14], the GigaTON database.

## Construction and content

### System design and implementation

Pipeline for *in-silico* mRNA expression levels, functional annotation, identification of assembly variants.

**Fig. 1:**
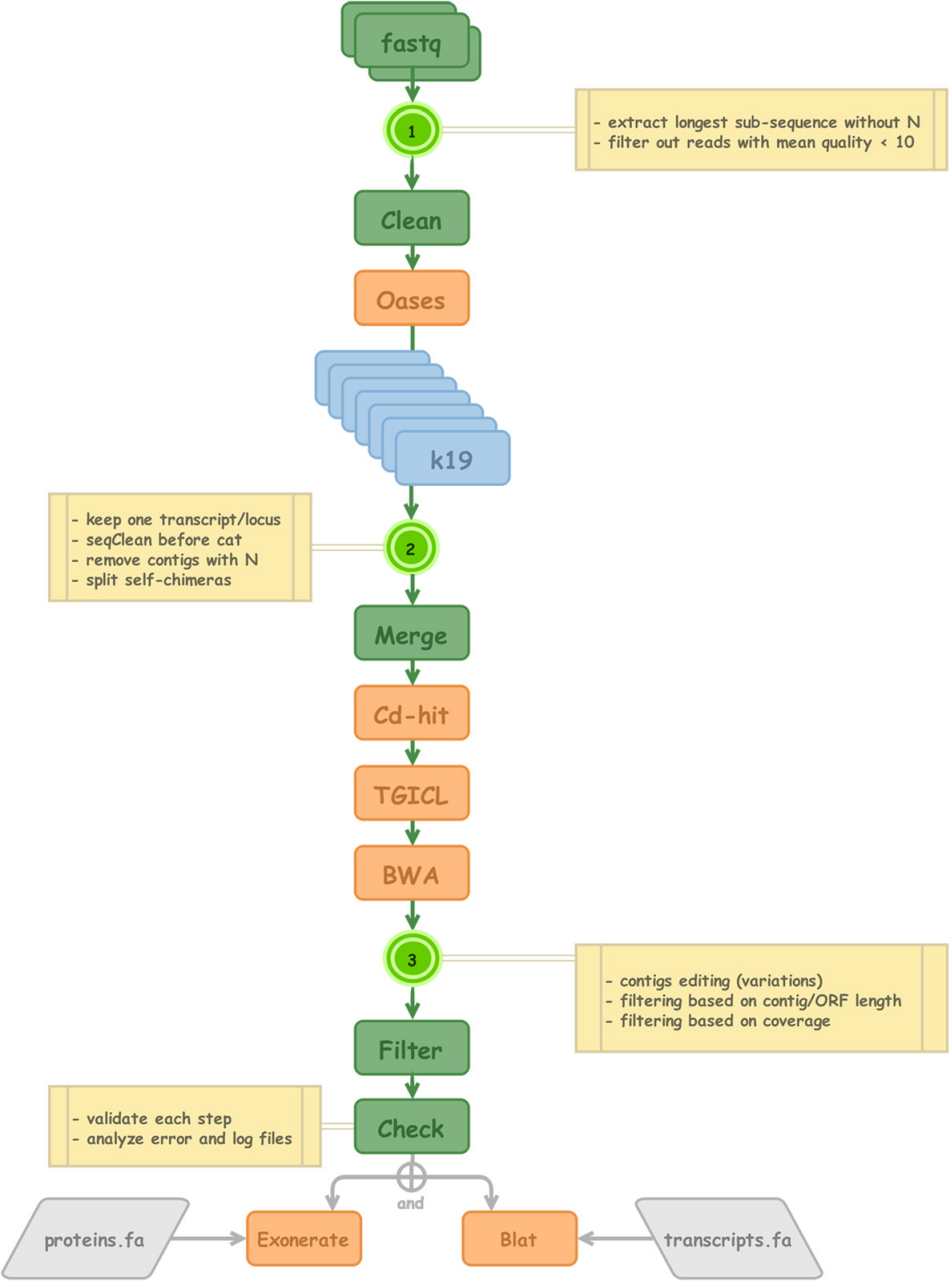
DRAP - Schematic representation of the *de novo* RNA-seq assembly pipeline structuring the GigaTON database. The fastq files from the RNA-seq libraries were filtered, cleaned and sorted according to their quality and the presence of undermined nucleotide content. Merged results were then edited for the existence of assembly variants and filtered according to the length and coverage. Results were compared to the existing proteins and transcripts

1. Data acquisition
  The 114 RNA-seq libraries were generated as a part of the oyster genome project [13], except for two of them that were used in a study dealing with salinity stress response [15]. They comprise 99 single and 15 paired raw read files and have been downloaded in fastq format, from the NCBI SRA website (study number SRP014559 and SRP013236, respectively).
2. Sequence/data cleansing
  The reads were first cleaned from remaining sequencing adapters using trim_galore (http://www.bioinformatics.babraham.ac.uk/projects/trim_galore/). Then the over-represented reads were filtered out using the normalize_by_kmer_coverage.pl script from the Trinity software package [16]. The next step aimed at discarding the invalid base calls by extracting the longest sub-sequence without unidentified nucleotides (Ns) from each read. If the length of the longest sub-sequence did not exceed half of the sequence length, the read and his mate, if present, were removed. This last step was performed using an in-house script (available upon request).
3. Contig assembly
  The assembly was performed in two steps described hereafter. A per sample assembly was first performed, allowing us to build a contig set for each sample. Then, all sample contig sets were merged, then duplicates were removed and references with very low read counts were filtered out.
  For each sample, nine Oases [17] assemblies using different k-mers (25, 31, 37, 43, 49, 55, 61, 65 and 69) were performed on cleaned reads. Each assembly produced a transcripts.fa contig file, which header includes an assembly locus. Only the longest and most covered (read depth) loci were kept using the OasesV0.2.04OutputToCsvDataBase.py script developed by a Brown University team (https://code.google.com/p/oases-to-csv/). After that, all files were merged. Finally, anti-sense chimeras (accidentally produced by the assembly step) were split or removed with an in-house script (available upon request).
  Because similar collections of contigs were produced by different k-mers, a cd-hit-est [18] step was used to remove redundant contigs based on their sequence similarities (parameters -M 0 -d 0 -c 0.98 -T 8). Because different kmers sometimes constructed different transcript parts, TGICL [19] was used to assemble contigs having significant overlaps (parameters -c 4 -l 60 -p 96 -s 100000)*.* The contigs were then filtered on a minimum length of 200 base pairs.
  Input reads were mapped back to the contigs using bwa aln [20]. The resulting alignment files were used to correct the contig sequences from spurious insertions and deletions using an in-house script (available upon request) and to filter out those with very low coverage. The filter excluded contigs with less than two mapped reads per million.
  All sample contig fasta files were merged and each contig renamed by adding the sample name at the beginning of its name. The longest ORF was then searched with getorf from EMBOSS [21]. A cd-hit clustering was performed on the ORFs with sequence identity equal or greater than 0.9 in order to extract from each cluster, the contig with the longest ORF or, if the ORF size are the same, the longest contig. The remaining contigs were clustered by cd-hit-est with sequence identity equal or greater than 0.95. Input reads from all conditions were then mapped back to the contigs using bwa aln [20]. Contigs with very low coverage, less than two mapped reads per million, were filtered out.
  The final set contained 56,651 contigs, corresponding to a total of 93,954,163 base pairs. The median and mean length of the contigs were respectively 1180 and 1,659.35 base pairs. The longest contig contained 66,407 base pairs (Fig. 1).
4. Functional annotation
  The contig set was annotated using blast versus several databases including uniprot-swissprot, refseq protein, refseq_rna, rfam, Ensembl oyster proteins and the contigs themselves.
5. Quantification
  All sample read sets were aligned on the final contig set using bwa aln to quantify the expression. A quantification table was built with these counts. It can be retrieved from the download section of the website (count_matrix.csv).
6. Variant calling
  The alignment files were processed with Samtools mpile-up. Variants including SNPs and INDELs were called using the following command line and parameters : samtools mpileup -EDu -d 10000 -q 30 | bcftools view -vcg | vcfutils.pl varFilter -d 6 -D 10000 -a 2 vcftools --maf 0.15. A subset of 336,018 variants was stored in the database and the complete variant files containing 2,144,528 SNPs and 77,274 INDELs can be found in the download section of the website. The polymorphisms have been annotated using snpEff v4.15 [22] using the transdecoder open reading frame annotations made on the contigs. The complete annotated VCF files have been added to the download page of the website, one for SNPs (ALL_SNP.oysterAssembly1.0_annot1.vcf) and another one for INDELs (ALL_INDEL.oysterAssembly1.0_annot1.vcf*)*. The results indicate that out of the 2,181,748 SNPs found, 33,98 % cause synonymous changes. The impact of SNPs and INDELS are detailed in Table 1 and Table 2, respectively.

**Table 1:** Impact of SNPs on the GigaTON protein set

TYPE (alphabetical order)	Count	Percent
3’-UTR variant	336790	16.308 %
5’-UTR premature start codon gain variant	36174	1.752 %
5’-UTR variant	220751	10.699 %
Initiator codon variant	52	0.003 %
Intergenic region	331008	16.028 %
Missense variant	431797	20.908 %
Missense variant + splice region variant	45	0.002 %
Splice regioin variant	1154	0.056 %
Splice region variant + synonymous variant	56	0.003 %
Start lost	323	0.016 %
Stop gained	3950	0.191 %
Stop gained + splice region variant	1	0 %
Stop lost	429	0.021 %
Stop retained variant	612	0.03 %
Synonymous variant	701868	33.985 %

The polymorphisms have been annotated using snpEff v4.15 [22] using the transdecoder open reading frame annotations made on the contigs. The complete annotated vcf files for SNPs can be downloaded from the GigaTON website (ALL_SNP.oysterAssembly1.0_annot1.vcf)

**Table 2:** Impact of INDELs on the GigaTON protein set

TYPE (alphabetical order)	Count	Percent
3’-UTR variant	32867	35.312 %
5’-UTR variant	12890	13.849 %
Chromosome number variation	15	0.016 %
Disruptive in-frame deletion	968	1.061 %
Disruptive in-frame insertion	502	0.539 %
Frameshift variant	5653	6.074 %
Frameshift variant + start lost	156	0.168 %
Frameshift variant + stop gained	84	0.09 %
Frameshift variant + stop lost	51	0.055 %
In-frame deletion	430	0.462 %
In-frame insertion	369	0.396 %
Intergenic region	39050	41.955 %
Start lost + disruptive in-frame deletion	3	0.003 %
Start lost + in-frame deletion	7	0.008 %
Start lost + in-frame insertion	1	0.001 %
Stop gained + disruptive in-frame deletion	1	0.001 %
Stop gained + disruptive in-frame insertion	1	0.001 %
Stop gained + in-frame insertion	7	0.008 %
Stop lost + in-frame deletion	1	0.001 %

**Fig. 2:**
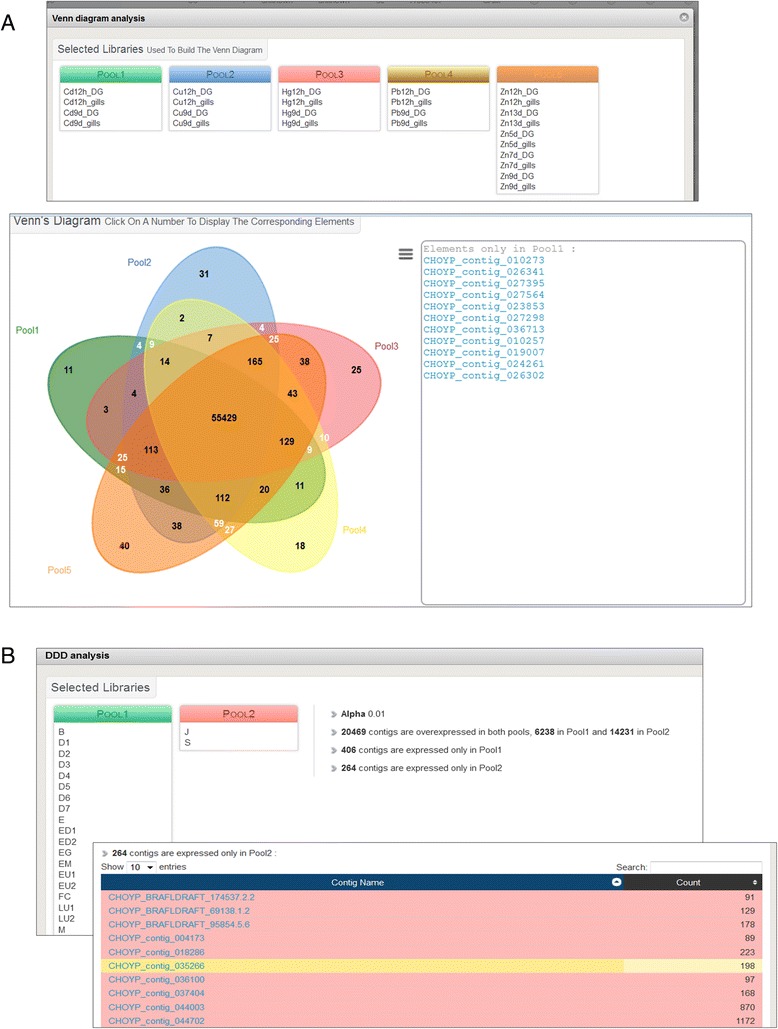
Screen captions illustrating online Venn diagram and digital differential display (DDD) analysis examples. **a**: search for the specific effect of exposure to cadmium among other heavy metals. Relevant sample libraries were pooled according to the metal but regardless of the exposure duration and the tissue (top panel). The resulting Venn diagram displays the metal-specifically overexpressed contigs as a ‘click-and-browse’ list (bottom panel). **b**: search for metamorphose-related transcripts. The sample libraries were pooled regarding the development stage in two groups separated by the onset of metamorphosis (top panel). The parameters and results of the DDD analysis are indicated on the right. Ten contigs out of the 243 that were only expressed after metamorphosis are displayed as a ‘click-and-browse’ list (bottom panel). All results can also be downloaded as .tsv files for further analysis
  Contig search and browser: the BioMart portal. The information about the contigs can be searched, browsed and retrieved online using the Biomart portal (www.biomart.org) [6]. Briefly, the GigaTON contigs or subgroup of contigs can be filtered out according to various criteria and sub-criteria (name, length, annotation number in a wide range of repository databases, Gene Ontology, keyword, expression), and retrieved with their corresponding attributes selected by the user among an extensive list of 71, including: filter criteria, best annotation identifier, hit start/stop position, number of variants, DNA sequence, expression pattern in tissues, developmental stages and various physiological conditions (Fig. 3). The retrieved contigs can be browsed further for additional information (sequence, counterparts in other databases, expression between libraries) in convenient and user-friendly point-and-click interface tabs (Fig. 4).

The INDELs have been annotated using snpEff v4.15 [22] using the transdecoder open reading frame annotations made on the contigs. The complete annotated vcf files for INDELs can be downloaded from the GigaTON website (ALL_INDEL.oysterAssembly1.0_annot1.vcf)
7. RNABrowse environment setting, database features and request tools
  Differential analyses: Venn diagrams and Digital Differential Display (DDD). The GigaTON database can be requested for differential analysis of contig content (Venn diagrams) and expression level between libraries (DDD) using a Fisher’s exact test. These requests are easily performed using a ‘point-and-click’ interface for sample library grouping, analysis parameters and results exploitation (browsing and downloading) (Fig. 2). A detailed description of each library can be found in the download section of the GigaTON website (libraries description .xlsx).

**Fig. 3:**
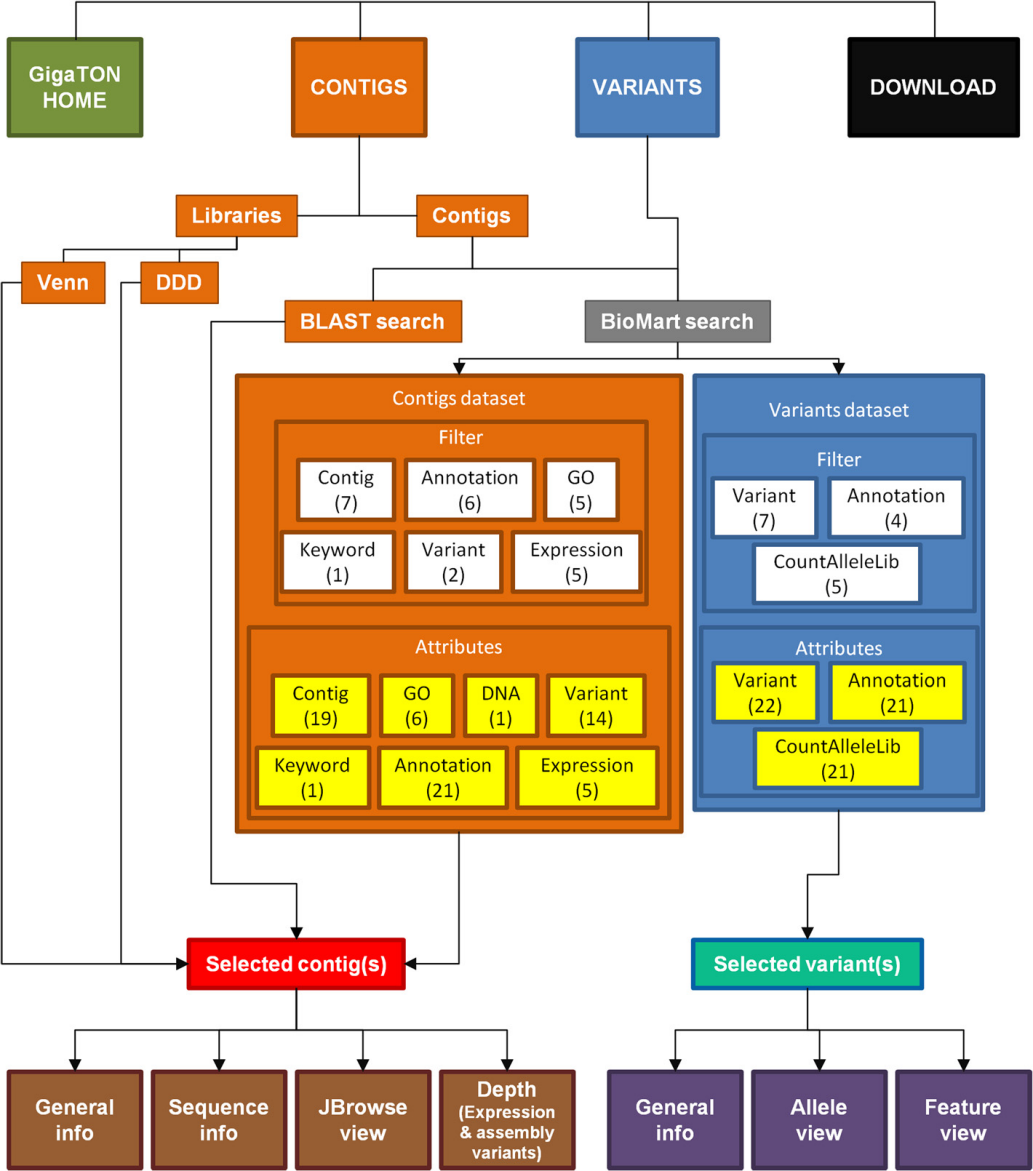
Schematic overview of the GigaTON pipeline search, browse and retrieve tool. The database can be used to investigate the transcriptome assembly by searching contigs (orange) or polymorphism variants (blue), or to download featured data sets and library BAM files (black). The search for a specific contig (or a contig subset) can be achieved either by differential analysis of library pools (Venn diagrams and Digital Differential Display (DDD)), either by BLAST or BioMart search. The BioMart portal (grey) enables to search a dataset using a combination of several filter criteria (shown in white), and to retrieve the results featuring a wide range of information characteristics and parameters, i.e. ‘attributes’ (shown in yellow). The resulting dataset (Selected contigs, red; Selected variants, shonw in turquoise) and associated attributes can be downloaded and/or browsed within the GigaTON pipeline for additional information. The latter include Sequence info (translation frames, ORF length…) or Depth (detailed view of expression level and assembly variants between libraries) for contigs (brown), or allele and feature views for variants (shown in purple). The number of user-exploitable fields/buttons is indicated in brackets under each corresponding criteria

**Fig. 4:**
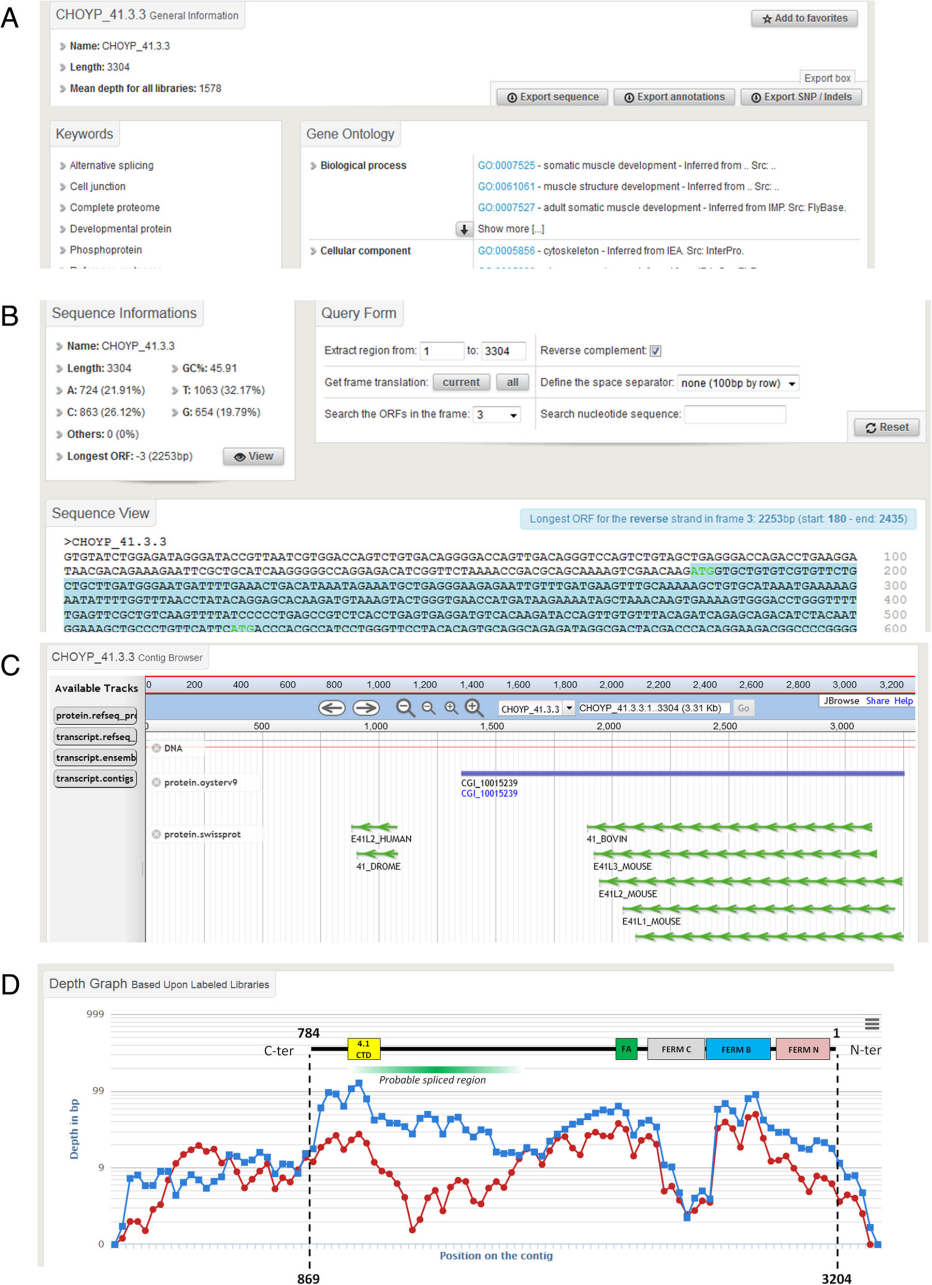
An overview of a contig search results. **a**: Caption from the ‘General Info’ tab. This screen enables the user to display and retrieve general information about the searched contig, including related keywords and functional annotation such as Gene Ontology. **b**: Caption from the ‘Sequence View’ tab. Statistics and DNA sequence of the contig are presented on this this screen. An embedded tool enables the user to search ORFs and get their translation. **c**: Caption from the ‘JBrowse View’ tab. This browser enables a quick overview of the contig and its corresponding counterparts in other databases. **d**: Caption from the ‘Depth View’ tab that contains expression data as the mean coverage and number of reads of the contig in all the libraries that can be chosen and displayed on a graph using a point-and-click interface. As an example, the contig CHOYP_41.3.3 is displayed in the Amu (in red) and Dgl (in blue) libraries. The structure of the corresponding protein has been added to the graph with the domain architecture. Discrepancies in mean depth coverage between library assembly variants may indicate splice variants at these positions (green gradient frame)

## Utility and discussion

### Gain of knowledge regarding existing databases

**Fig. 5:**
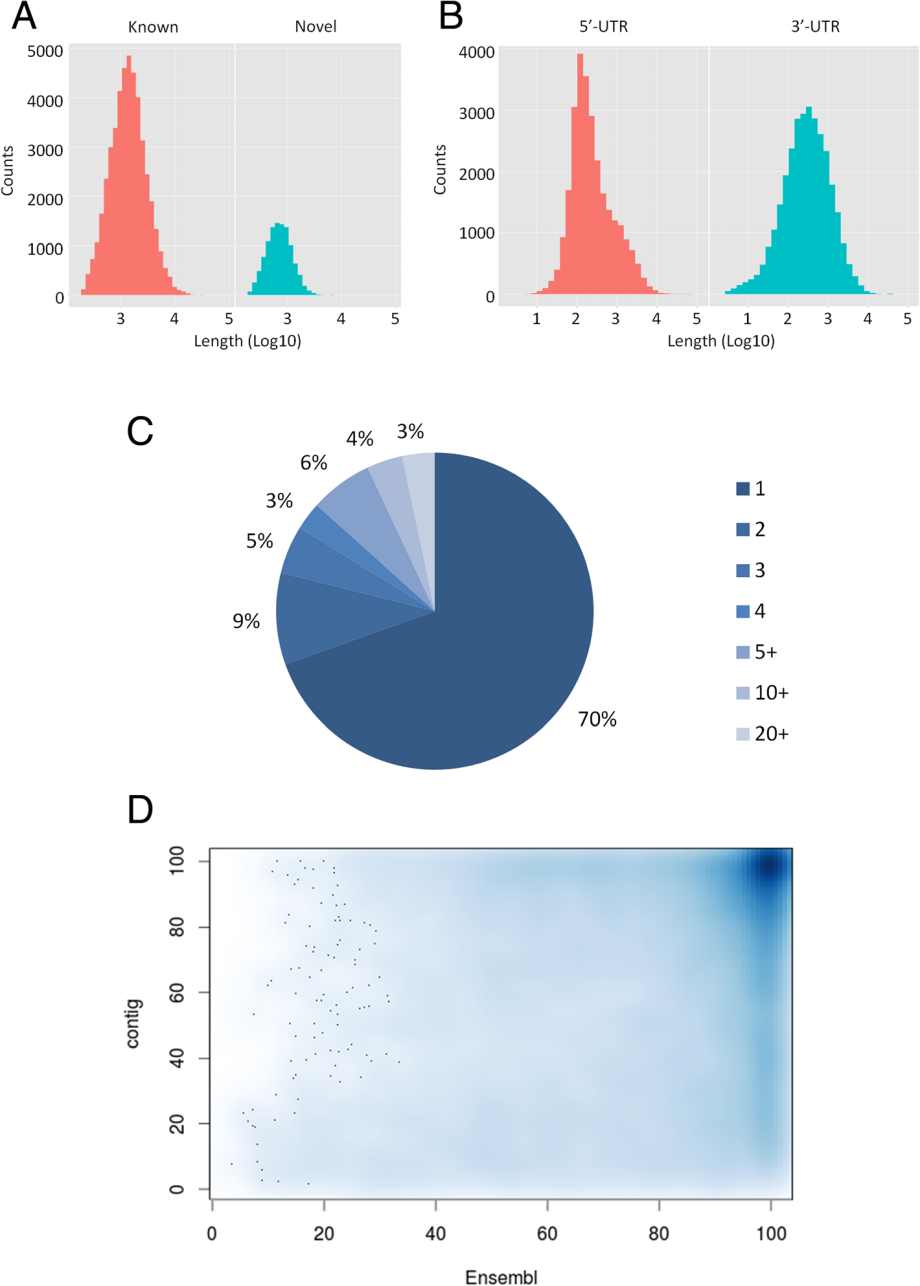
Contribution of the GigaTON database. **a**: Length distribution histogram of the known (red) and the ‘novel’ (blue) contigs. **b**: Length distribution of the newly characterized 5’- (red) and 3’- (blue) UTRs. **c**: Distribution of assembly variants in the database. The graph indicates the proportion of contigs exhibiting the indicated number of variants. **d**: Correspondence between the GigaTON and the predicted geneset proteins. The GigaTON contigs were translated using TransDecoder (http://transdecoder.github.io/) and resulted in 41445 proteins. They were aligned with the Ensembl protein from the oyster genome. The relative length of the 16787 alignments displaying at least 95 % identity between sets was plotted. The vertical smear on the right reflects longer proteins in the GigaTON database. For example, the protein with coordinates (X = 100, Y = 50) has its full Ensembl sequence covering 50 % of its GigaTON counterpart

In the present work, we generated 56,621 high quality contigs. This assembly brings an important amount of additional information when compared to existing databases (+52.3 % in contig number compared to the EST library GigasDatabase v9 [1]). This number is also greater than the 28067 consensus genes that are predicted in the oyster genome [13]. This important increase in the transcript sequence information does not only lie in the number of contigs (Fig. 5a) but also brings the 5’ and 3’ UTR information (Fig. 5b). Indeed, even though 454 sequencing was performed on cDNA from various oyster samples [13], none of the corresponding assembled full-length transcript sequence is, to our knowledge, publicly available to date. Therefore, we believe that the GigaTON database constitutes the new reference trancriptome in the Pacific oyster, a work of significance for the community. Some of its most relevant features are developed below.

First, the novelty and gains of GigaTON are novel contigs. Among the 56621 contigs in GigaTON, 53,515 contigs (94,5 %) align to the Ensembl Oyter genome, with a vast majority displaying 95 % identity and 90 % coverage. A contig can be considered as ‘novel’ when its sequence is unknown or present in databases but not annotated, therefore including those which do not align with the genome, in addition to those that align with unannotated areas of the genome. The GigaTON database contains 38,316 contigs (67.7 %) that display an overlap with one of the 28636 genes contained in the oyster Ensembl GTF file. The remaining 18305 ‘novel’ contigs have been aligned to the RefSeq protein database, (http://www.ncbi.nlm.nih.gov/genome/annotation_euk/Crassostrea_gigas/100/) which contains more transcripts (45,394 gene models). Out of the 18,305 ‘novel’ contigs, 7750 produce significant alignments with a RefSeq protein. Among these contigs, 2241 have no hit on the Ensembl oyster genome and 1779 match oyster proteins in the RefSeq database, whereas the remaining match known proteins in other organisms. More drastically, 11,817 ‘novel’ contigs within GigaTON did not match any annotated region of the genome nor any oyster RefSeq protein, whereas 1078 have a RefSeq annotation in other organisms, mainly *Lottia gigantea*, *Saccoglossus kowalevskii*, *Hydra vulgaris*, *Nematostella vectensis*, *Branchiostoma floridae* and *Strongylocentrus purpuratus* (139, 83, 76, 72 62 and 52 hits, respectively). The distribution statistics of the length of the ‘known’ vs. ‘novel’ contigs concerning the coding regions are : minimum, 200 vs. 202; 1^st^ quartile, 821 vs. 500; median 1372 vs. 730; mean, 1860 vs. 899; 3^rd^ quartile, 2258 vs. 1070; maximum, 66410 vs. 19500, respectively (Fig. 5a).

An example of a novel contig in GigaTON is the contig ‘CHOYP_contig_047748’ that corresponds to the sequence encoding the precursor of a novel oyster neuropeptide family named “RxIamide”. This sequence does not match any oyster Ensembl predicted gene, cDNA or protein, despite (i) ESTs present identity (HS206944, HS234774), and (ii) the gene products were characterized using mass spectrometry [23]. However, the considered sequence matches unannotated genomic sequences in the scaffold 40412 (43,230-43,117; 30,085-29,480). This situation is not unique and was also met for a number of neuropeptide-encoding genes (Buccalin, cerebrin, GGN-related peptide, LRNFVamide, opioid neuropeptide-like, tachykinin-like) [23]. Such examples illustrate the gain brought by GigaTON database, which, in addition to bringing new transcriptomic information, might help improving gene prediction.

Second, the GigaTON contigs provide insights into the 5’ and 3’ untranslated regions (UTRs) of the oyster transcribed sequences (Fig. 3 and Fig. 5b). Importantly, the 5’ information is a significant asset towards the determination of a consensus promoter sequence in a bivalve mollusk, which may help unlock functional genomics studies in lophotrochozoans. Besides, there is a widely admitted approximation that considers the 1 kb sequence upstream translation starts as being proximal promoter sequences, which is commonly used at present for the interpretation of NGS data [24–26]. However, the mean 5’-additional information we provide in the GigaTON database is around 600 pb (Fig. 5b), indicating a mean 600 pb length for 5’UTRs in the Pacific oyster. Therefore the aforementioned approximation presents a potential severe bias since up to 60 % of the sequence information might be mis-considered as promoter sequence instead of UTR, and thereby should be cautiously considered, as illustrated for some *Hox* genes orthologues [27]. This contradicts the general assumption that oysters, like other lophotrochozoans, have very short 5’-UTRs. However, because the length of the predicted proteins corresponding to the GigaTON contigs also displays a great increase (Fig. 5d), the additional 5’ sequence information can also correspond to coding DNA, thereby minimizing the flaw in proximal promoter approximation previously stated. The same rationale can be proposed regarding the additional 3’ sequence information provided within the GigaTON contigs as being 3’UTRs and/or coding regions. This point is important for the understanding of the mi/piRNA pathway in oysters, which mostly affects 3’-UTRs in vertebrates [28, 29] but could also highlight alternative polyadenylation signals in mollusks. Therefore, additional work, including functional analyses dedicated to the determination of consensus promoter and/or polyadenylation signals, are required to decipher such potentially important issues for our understanding of gene expression regulation in the Pacific oyster, and should take great advantage of the present database.

Second, the GigaTON pipeline led to the identification of a high number of assembly variants, since 30 % of the contigs display at least one variant (Fig. 5b). Some of these assembly variants highly likely correspond to transcript variants that could reflect alternative splicing when these variations are located within ORFs. Alternatively, when variations are located within UTRs, the presence of transcript variants could illustrate the usage of alternative promoters and/or polyadenylation signals. Both can have important functional outcomes in terms of protein structure or gene expression level regulation. As an illustration, the contig CHOYP_41.3.3 (Fig. 4) exemplifies a putative transcript variant that might lack a region between two functional domains, thereby diminishing the length of the protein with probable physiological consequences.

Third, the great increase in length for a number of proteins as mentioned above, points out that the *in silico* protein prediction from the genome, which is mostly based on homology with other invertebrates, is significantly improved by the present assembly. This indicates a noticeable difference in protein sets between the Pacific oyster and other invertebrates. Such a discrepancy between invertebrates seems surprising, except if one considers the growing number of documented lophotrochozoan features, such as DNA methylation patterns [24, 30], angiotensin-converting enzyme characteristics [31, 32] or BMP cDNA sequences [33], that display a closer resemblance with vertebrates than with ecdysozoans. Besides, in a similar fashion than mapping long 454 reads helped assembling genomic scaffolds [13], the GigaTON database provides even longer contigs that we think could importantly help improve the current assembly of the oyster genome. These three features might be of functional significance for our understanding of the oyster physiology and support the importance of the GigaTON database, which encompasses a high diversity of transcriptomes, in the context of post-genomic studies in lophotrochozoans.

### Easiness, potency and versatility of the tools

The GigaTON database provides, to our knowledge, the most complete and integrated transcriptomic resource to date in a marine mollusk. A remarkable strength of this database is the possibility to browse data quickly and easily to get a global picture of the user’s question using Venn diagrams or DDD, which can easily be further examined in deep details. This is noticeably allowed by the use of the BioMart portal within GigaTON, which links extremely massive sequence and expression data in various physiological conditions with a user-friendly, easy to use, point-and-click interface. Our searchable online database enables a high versatility tool both in terms of request criteria and retrieved attributes that can be easily downloaded and further analyzed using dedicated software packages. In parallel, specific contig characteristics such as transcript variants, polymorphism and expression can be browsed online in the graphic interface. Furthermore, the included browser allows a convenient view of the target contig counterparts in numerous other databases at a single glance (Fig. 4c), without any particular need for the user to be skilled in bioinformatics. Therefore, the GigaTON database is most likely to be extensively accessed by an increasing scientific community studying evolutionary, environmental or aquaculture related aspects in *C. gigas*. Together with the current genome assembly, it constitutes one of its strong assets as an emerging model species.

## Conclusions

In this work, we assembled 114 RNA-seq libraries, mostly generated aside of the oyster genome project, into a publicly accessible database that includes user-friendly ‘search, browse and retrieve’ online tools, the GigaTON database. To date, the GigaTON database constitutes the most extensive transcriptomic resource in a marine invertebrate to our knowledge, and sets the new reference transcriptome in *C. gigas*, one of the most important shellfish aquaculture resources worldwide.

## Availability and requirements

The GigaTON database is available at http://gigaton.sigenae.org. There are no restrictions for its use by non-academics.

## Abbreviations

EST: expressed sequence tag
ORF: open reading frame
Ns: undetermined nucleotides
UTR: untranslated region
